# Against the proportionality principle: Experimental findings on bargaining over losses

**DOI:** 10.1371/journal.pone.0218805

**Published:** 2019-07-22

**Authors:** Wulf Gaertner, Richard Bradley, Yongsheng Xu, Lars Schwettmann

**Affiliations:** 1 Department of Economics, University of Osnabrück, Osnabrück, Germany; 2 CPNSS, London School of Economics, London, United Kingdom; 3 Department of Philosophy, Logic and Scientific Method, London School of Economics, London, United Kingdom; 4 Department of Economics, Andrew Young School, Georgia State University, Atlanta, Georgia, United Sttaes of America; 5 Helmholtz Zentrum München GmbH, German Research Center for Environmental Health, Institute of Health Economics and Health Care Management, Neuherberg, Germany; 6 Martin Luther University Halle-Wittenberg, Halle, Germany; Hanken School of Economics, FINLAND

## Abstract

The outcomes of bargaining over losses, the subject of this paper, have rarely been studied. But experimental studies of related situations, such as those involving bankruptcies or bequests in which the sum of the legal claims that can be made against a bank or firm or estate are greater than their values, have produced strong support for the proportionality principle. To test whether this principle would find support in other situations involving losses we designed an experimental game in which four players start out with differing initial endowments of real money. They are then informed that a certain amount of this resource has to be given back to the experimenter. How should the loss be shared among the agents? This game was run at different locations and under different treatments over a period of almost three years. We found that the proportionality principle was rarely proposed and even less frequently accepted as a solution to this problem. One of the main reasons for this result was that the two players with the smallest endowments opposed most of the proposals which asked them to contribute at least some positive amount of their own initial resource.

## Introduction

Bargaining situations are ubiquitous. Wage negotiations between a group of employers and a trade union or trade agreements between single countries or between larger associations, both political and geographic, immediately come to mind. In such cases, agents or nations get together in order to jointly bring about an outcome, superior to the initial status quo, which each of the participants would not have been able to materialize just by itself. This surplus is then the object of bargaining. Various theoretical solutions have been proposed in the economics literature [*1–5*].

The outcome of bargaining over losses, the subject of this paper, has rarely been investigated. Experimental studies of related situations, such as those dealing with bankruptcies or bequests, have produced strong support for the proportionality principle as the appropriate sharing rule. Would this principle find support in other scenarios involving losses as well? To find out, we designed an experimental game in which four agents start out with differing initial endowments of real money. These are assigned to the players either via a random mechanism or, in a different treatment, in relation to the success in a real effort task (knowledge quiz). The agents are then informed that a certain amount of the initial resource has to be handed back to the experimenter. How would such an enforced loss be shared among the players? We find that the proportionality rule only plays a minor role in our scenarios. Proportionality is proposed relatively often in early stages of the games by some of the players but is hardly ever accepted unanimously by all members of the same group. The two players with the smallest endowments in particular often refuse to contribute at least some amount of their own initial resource.

Closest to what we shall focus on, though substantially different in character, are situations of bankruptcy and bequest to which we briefly referred above. In cases of bankruptcy, various agents who are depositors or shareholders have *legal claims* of differing magnitude against a bank or company, and the fundamental question is how the liquidation value which in total is smaller than the sum of claims, should be distributed among the claimants. In a bequest situation, the value of the estate left behind by the head of a family, let’s say, is not large enough to honor the titles to legacy or the informal promises that had been made by the testator. For these problems, several solutions have been suggested, among them the proportional rule, the egalitarian principle, the constrained equal awards and the constrained equal losses rule [*6–11*]. These rules are studied theoretically, together with a recent survey of the literature, in [*12*]. The constrained equal awards rule is such that awards are made as equal as possible under the constraint that nobody receives more than his or her original claim. The constrained equal losses rule divides the amount that is left in such a way that the losses incurred by all claimants are as equal as possible subject to the condition that no one receives a negative amount. The egalitarian principle demands an equal split of an available resource, let’s say, while the proportionality principle requires a division in proportion to legal claims or financial status or work effort.

We think that situations in which losses occur or may occur are not restricted to cases of bankruptcy or bequest, in which legal claims quite often are most decisive. It is in this sense that we view the scenarios we shall focus on as being substantially different in character.

Some reference to real-world phenomena may help to make our position more understandable. The collapse of Lehman Brothers in 2008 generated a severe financial crisis in many Southern European countries which forced governments to introduce a policy of austerity with the consequence that larger parts of the population in these countries had to put up with cuts in wages, old-age retirement benefits, health coverage and other payments that had been granted over many years. The decision of a majority of the voters in the UK to leave the European Union will introduce economic losses on either side according to what many experts prognosticate. The annihilation of multilateral trade agreements between the US and groups of countries both in the Western and Eastern hemisphere by the current President of the United States may have similar consequences. A large-scale migration of refugees from Africa, Asia and the Near East may induce severe costs for the population in central Europe. How this burden will eventually be shared appears to have turned into a highly controversial matter among the European nations. All these contexts may add further aspects and insights, which are not covered by situations of bankruptcy or bequest.

Losses, in particular pecuniary losses, are not just the reverse side of the coin of monetary gains. Losses lower an achieved status quo to which agents are highly sensitive whereas gains are viewed as a welcome addition to the present state, an asymmetry similar to what has been experimentally verified in situations of risk [*13*]. For situations of bankruptcy in particular but also for salary negotiations or pension claims, there are a limited number of questionnaire results as well as findings from experimental games [*14–19*]. In questionnaire experiments, outside observers or agents in the role of an arbitrator make their decisions overwhelmingly in accordance with the proportionality principle (see in particular [*14–17*] whereas in experimental games the results with respect to the four rules defined above are mixed. In a coordination game based on the majority principle, the proportionality rule again gained wide support [*17*]. This rule, however, clearly trailed behind equal split and constrained equal awards in actual negotiations, namely free-form bargaining with asymmetric claims [*15*]. To be more specific, those negotiations which were held between two agents showed that constrained equal awards gained more support than equal split when the difference in claims was “smaller”, while the opposite was the case when the difference in claims became “larger”. On the other hand, in [*19*] it was found that a wide majority of the participants showed “striking support”, as the authors write, for the proportionality rule when they acted as third-party arbitrators in bankruptcy situations in which agents had acquired different claims through work, before the firms had gone bankrupt. In the latter laboratory experimental setting, arbitrators also significantly supported the equal losses rule, while the equal awards principle received hardly any support. In [*18*] subjective entitlements derived from a production process were found to be clearly skewed toward the better performers when information about the individual performance was available.

We should add that there also exists a literature on economic laboratory experiments regarding the distribution of losses or benefits. In several cases, two-person ultimatum games have been staged to compare situations of gains and losses or two-person Nash demand games are played between agents [*20–22*]. The study in [*22*], for example, revealed in an ultimatum-game context that outcomes that are close to equal split are more likely to be observed in the loss domain than they are in the gains domain. This result is mainly due to a behavioral change on the part of the proposer who demands less in situations of losses than in cases in which bargaining over gains takes place, with the consequence that the payoffs the responders receive are higher under losses than they are in gain situations. Clearly, the setting in these experiments is different from ours. A two-person ultimatum game, and the emphasis is on “ultimatum”, has different characteristics than our free-form bargaining situations involving four players. Also, there is no explicit role for arbitrators in our set-up, as is the case in [*23*], where the authors ask how past experiences in the role of a rich or poor stakeholder influence the impartiality of arbitrator decisions and furthermore, how past experience in the role of an impartial arbitrator influence stakeholder behavior in their stakeholder role. Among other results, it is found that stakeholder allocations with past arbitration experience differ more from the impartial ideal than the stakeholder allocations by agents without arbitration experience. Two investigations, which are again somewhat closer to our own set-up are [*24–25*]. In [*24*], a Baron-Ferejohn model of multilateral legislative bargaining [*26*] is examined. In the gains version, three players decide how to split a given sum of money under the majority rule. In the costs version, players must decide how to raise funds to pay for a common project. In the gains version, the amount to be split decreased, in the costs version, the amount required for the project increased, when the proposal of the previous round got rejected by majority vote in the current round. In one series of experiments, there was stronger proposer power in the costs sessions, with relatively low proposer shares rejected significantly more often under gains than under costs. In [*25*], a multilateral bargaining game was studied in which five committee members decided how to distribute the benefits reaped from a common project. The experimental evidence in this investigation tends to support the proportionality rule: higher contributors receive higher shares of the benefits.

After taking all these different questionnaire and experimental-game based contributions to gain and loss division into consideration, it is perhaps justified to assert that the proportionality principle plays a prominent if not dominant role in agents’ decisions. This rule can be traced back at least to Aristotle in his Nicomachean Ethics [*27*]. Aristotle wrote: “What is just…is what is proportional, and what is unjust is what violates the proportion. So one share becomes too large and the other too small. This is exactly what happens in practice: the man who acts unjustly gets too much and the victim of injustice too little of what is good.” Interestingly, the proportionality principle also plays a major role in constitutional or human rights law [*28*]. According to our knowledge, it is the most applied rule in liquidation law in democratic societies [*29*]. It is neutral among claimants in the sense that neither large nor small claims are particularly favored in the distribution of the liquidation value. It prevents manipulability in so far as there is no incentive among creditors to merge or split their claims [*30, 31*]. The proportional rule has also been shown to be a part of any pure strategy subgame perfect Nash equilibrium in which the value of an estate is endogenous, depending on agents’ investment decisions [*32*]. Investment behavior is also the issue in [*33*]. It is experimentally shown that replacing the proportionality principle with the equal losses (equal awards) rule increases (decreases) the creditors’ total investment.

In questionnaire studies on burden sharing at several Southern European universities after the collapse of Lehman Brothers, run by two of the present authors, it was found that the proportionality principle lost much of its attractiveness among the students, once progressive modes were offered as alternatives which put smaller burdens on lower and larger burdens on higher income brackets [*34*]. These results prompted us to conceive an experimental game of loss sharing and to see how the proportionality rule would fare in an incentivized and highly controlled environment, which allows us to filter out the effects of contextual factors related to any real-world example of loss-sharing. As already briefly stated at the beginning of the introduction, the main finding of our investigation is that proportionality was proposed very rarely by the players and was accepted as a final solution in even fewer cases. This is in stark contrast to several of the experimental results referred to above.

## The experimental set-up

The detailed protocol of our game on loss sharing and the complete instructions given to the participants can be found under S1 File in the Supporting Information. We ran our experiments at five different places in Europe. In each experimental session, participants are assigned to a group of four individuals. Starting from an initial distribution of endowments in terms of real money (Euros and British Pounds, respectively), a loss has to be shared. A total amount of 50 Euros (Pounds) is distributed randomly to the four participants in each group where the assignment vector is (5, 10, 15, 20). The players are informed that a total amount of 10 Euros (Pounds) has to be handed back to the experimenter.

One randomly chosen member within each group then has two minutes to make a first proposal of how to distribute the loss of 10 Euros (Pounds) among the members of this group. Proposals that lead to individual losses higher than a person’s initial endowment are not accepted. After this first proposal, the other members of the group are asked to either accept or reject the proposal. If the proposal is accepted by *all* members of the group, the experiment is over for these four players. The participants were informed that failing to provide a rejection or an acceptance to a proposal by some other member in the group within one minute would be deemed as acceptance. As a matter of fact, there were only 11 instances of “silence” across all sites and all 686 rounds that were played.

Should there be no agreement after the first round, i.e. at least one of the three other group members objects, a second person in this group, again determined by chance but, of course, different from the first, will have to make a proposal of how to split up the loss of 10 Euros (Pounds). As long as no agreement on some proposal is reached among the group members, a new person is randomly chosen to propose a distribution of the loss.

Whenever at any point in the game, one proposal receives unanimous agreement, the experiment is over. There is a 20-minute time limit for each experiment. If after 20 minutes, no past proposal received unanimous support, the experiment is terminated automatically. In this case, a random mechanism will pick one of the past proposals as the final decision on the share of the loss.

At this point, we would like to comment on specific features of our bargaining game. First, in contrast to related experiments [*14*, *15, 19–22*], we consider games with four rather than two participants as is typical of dictator and ultimatum games. This offers a richer set of potential distributions. The second feature is the issue of unanimity. If unanimous consent were not required, the method of simple majority decision or some related rule could bring about an exploitation of the fourth player, possibly but not necessarily the weakest or the strongest in terms of initial endowment. Experimental evidence in [*35*] clearly supports this view. Under majority voting, in a group of three agents, all the loss was heaped on one member, in a group of five, either one agent had to carry the total loss or two members had to bear one half each. Such aspects might have led to different research questions which we do not want to consider here [*24*–*26*].

There is a third feature which is more crucial. What should the solution of the bargaining game be if after 20 minutes there is no unanimous agreement? In our base version, we opted for a random mechanism that would pick one of the proposals made within the twenty-minute time limit. This ending is quite different from protocols which declared that participants would earn nothing except for a show-up fee, in case they were unable to reach an agreement within a given time period [*14, 36*]. Our mechanism can, of course, induce players to propose a loss assignment, which is particularly favorable for them, and continue to repeat such a proposal whenever they have been selected to make the next proposal.

Therefore, we decided to introduce in our later experiments (at the London School of Economics in November 2017 and March 2018) another treatment of the case when there was no agreement within a group after 20 minutes. We informed the players in this version that in case of no agreement the experimenter would take a decision on his or her own in order to resolve the bargaining dispute. In this treatment, the players may start pondering what the experimenter might dictate. Would it be equal split of the loss, a proportional share or some other division? Would either of these imagined solutions then be a reference point in the bargaining procedure? Clearly, once this path is taken, an additional element enters, namely expectations towards fairness or something else on the part of the experimenter. However, by comparing these two different endings, we are able to investigate potential differences. The situation here is similar to the “chilling” effect of final offer arbitration [37]: Binding arbitration lowers costs of dispute relative to other options, and as a consequence, the disputants may rely more on arbitration and less on negotiations to settle controversies.

Furthermore, in our later experiments in London, we considered another variant of our base game. The random assignment of endowments at the beginning of each game was replaced by a real effort situation, i.e. the personal result in a knowledge contest. In other words, the assignment of initial endowments was determined according to the success in a quiz (see S1 File in the Supporting Information for details). Here, our hypothesis is that respondents might be willing to put a larger burden on those with a lower endowment if the corresponding amount has been “earned” by those persons, just as evidenced in the legislative bargaining experiment conducted in [*25*].

We ran our experimental game on loss sharing between April 2015 and March 2018 at the Technical University of Berlin, the University of Ireland in Galway, the University of Halle-Wittenberg, the University Carlos III in Madrid, and the London School of Economics (LSE). This allows us to control for potential local effects. Table 1 provides an overview of all sessions and the corresponding treatments. The students who participated in these experiments were enrolled in graduate or undergraduate programs, either in economics or in business administration, very few in political science, so that we obtained a fairly homogeneous group of players. All in all, we had 90 groups of four players each. We should mention in passing that we staged five constellations at the universities of Berlin and Halle with an equal endowment among all players within a group. In such a situation, it would be strange to expect anything different from an equal share of the loss (and this was actually achieved very quickly in four of the five cases, thus supporting similar findings in [*35*]), but we wanted to confirm that the general setting of the game is coherent and that departures from equal share are mainly due to an unequal initial endowment.

**Table 1:** Overview of locations, treatments, and proposal of proportional division.

Place [Sample]^a^	Endowments based on:		Decision in case of no agreement:		Number of groups	Total number of all proposals ^b^	Number of proposals of proportional division ^b^			Mean fraction of proposals of proportional division within each group ^b^
	Random assign-ment	Quiz results	Random mechanism	Experimenter decides			First round	All rounds	Accepted as final solution	
Halle [1]	x	-	x	-	6	40	1^b^	9	0	0.1660
Madrid [2]	x	-	x	-	6	44	1	5	1	0.1141
Galway [3]	x	*-*	x	*-*	7	54	3	7	1	0.2718
Berlin [4]	x	-	x	-	4	46	1	7	0	0.0771
Halle [5]	x	-	x	-	7	53	1	2	0	0.0804
London [6]	x	-	x	-	8	82	1^b^	5	0	0.0870
London [7]	x	-	-	x	21	114	4	11	1	0.1335
London [8]	-	x	-	x	14	89	7	14	1	0.2268
London [9]	x	-	-	x	7	46	2^b^	8	2	0.2806
London [10]	-	x	-	x	10	107	4	11	2	0.2308
Sum (% of total)					90	675	25 (28.7%)	79 (11.7%)	8 (8.9%)	

^a^ Experiments were run at the Martin Luther University Halle-Wittenberg ([1] April 30, 2015, [5] June 23, 2016), the University Carlos III in Madrid ([2] October 21, 2015), the University of Ireland in Galway ([3] November 26,2015), the Technical University of Berlin ([4] January 28, 2016), and the London School of Economics ([6] July 5–6, 2017, [7] and [8] November 20–21, 2017, [9] and [10] March 1–2, 2018).

^b^ Cases in which the proposer did not make any proposal within the two-minute time limit are excluded. There was no first-round proposal in one group in Halle [1], London [6] and London [9], respectively.

All analyses that follow have been conducted with the software package STATA^TM^ (version 14). Both the programming and the realization of the experiment were done with the web-based “Software Platform for Human Interaction Experiments (SoPHIE^TM^)” offered by SoPHIE Labs (www.sophielabs.com).

## The proportionality issue: Results

The proportionality principle can be viewed as a device of compromise. Given the initial endowments (5, 10, 15, 20) underlying our experiments, it implies losses of (1, 2, 3, 4) for players 1 to 4, respectively, and thus treats all income receivers equally in the sense that the burden or loss that it demands hurts all groups the same *relative* to the amount that they possess or earn. In contrast, an equal split of the loss, namely (2.5, 2.5, 2.5, 2.5), affects all groups of income receivers equally in *absolute* terms but, of course, hurts the lower incomes in relative terms very severely whereas the higher income brackets remain relatively unscathed. The proportionality rule can be seen as lying between the constrained equal awards rule, which is more beneficial to the lower groups, and the constrained equal losses rule, which burdens the lower income receivers very severely but is favorable to the higher brackets. We should mention that in some axiomatic work a “compromising rule” between proportionality and the constrained equal awards rule and between the former and the constrained equal losses rule was proposed [*38*].

Table 1 above documents the occurrence of the proportionality principle as a proposal in the very first round, in all rounds of all groups, and as an accepted division at all locations where we ran our game of losses. Contrary to findings particularly in [*17,19*] to which we referred in our introduction, proportionality was suggested as a solution surprisingly *rarely*, across all locations and across all treatments. Let us start with the overall descriptive picture. There were 87 first-round proposals and 675 proposals in all rounds concerning all 90 groups. Proportionality was initially suggested in 25 groups (28.7%) and proposed at 79 (11.7%) instances, of which in turn, eight received unanimous agreement. Hence, if proposals of a proportional division are made, this occurs rather early in the bargaining process.

Next, we focus on potential treatment effects. To do this, we calculated fractions of proposals in accordance with proportionality at the group level (see S1 Table in the Supporting Information for corresponding fractions for all groups). The last column in Table 1 reports mean values of these fractions for the different samples. To compare differently treated samples we conducted two-sample Wilcoxon rank-sum tests using the fractions at the group level as units of observation. First, to investigate the effect from two different tools applied to assign endowment levels we compared “random assignment” (samples [7, 9]) and the “quiz” (samples [8,10]) cases in London. The two mean values of corresponding fractions (0.1703 and 0.2285) are rather close to each other and the two-sample Wilcoxon rank-sum test does not reveal any significant differences (p = 0.2938). Therefore, the fraction of proposals for a proportional division does not significantly differ between the two treatments so that we pool these samples in the second step. When we compare the “random mechanism” and the “experimenter decision” in case of no agreement, the mean values of the fractions of proposals for proportionality differ somewhat (0.1355 vs. 0.1971) but, again, the two-sample Wilcoxon rank-sum test does not indicate relevant differences (p = 0.3959). Hence, also in this case the proposal of a proportional division does not depend on either of the two decision mechanisms in case of no agreement.

Furthermore, at each individual location, the occurrence of proportionality as a proposal in relation to the total number of proposals at that site is rather low and ranges between 3.8% (sample [5]) and 22.5% (sample [1]). The small number of groups comprising several samples prevents us from conducting further statistical tests, but we could not detect any significant differences between the groups in London compared to those from other places (p = 0.6015). However, even more notable is the rejection of the proportionality rule as a final outcome. At most locations, the absolute number of cases of acceptance of this rule is either nil or one.

Besides different treatments and locations, it is to be expected that the initial endowment of each player influences both his or her decision to propose a proportional distribution and the decision to accept or reject a corresponding proposal by another player. Part a) in Table 2 examines the 79 proposals of a proportional allocation in all rounds and states which player made a proposal and which players subsequently rejected it. The total number of proposals by each of the four players across all groups is roughly the same. However, players 1 and 2 propose a proportional share much less frequently than players 3 and 4. Interestingly, players 1 and 2 hardly differ in terms of the number of proposals for proportionality. The same observation is true for players 3 and 4. The difference between the relative frequencies of corresponding proposals for the two pairs of players (viz. 27/335 = 0.081 vs. 52/340 = 0.153) is statistically significant at the 1%-level according to a two-sided Fisher’s exact test (p = 0.004). Similarly, the two players with the lowest endowments are more likely to reject a proportional allocation. While player 1 rejects 49 out of 64 proposals made by others (i.e. 76.6%) and player 2 rejects 47 out of 67 (70.1%), the corresponding values are 15 out of 51 (29.4%) and 9 out of 55 (16.4%) for players 3 and 4, respectively. To evaluate the significance of these differences we conducted several two-sample tests for proportions which confirmed that the relative frequencies of rejections of proposals for proportionality do not differ between players 1 and 2 (p = 0.4070) and players 3 and 4 (p = 0.1088), respectively, but are significantly different at the 1% level between (all combinations of) players from these two groups. Apparently, many players with the lowest endowment consider a proportional burden as too much to accept. It is then understandable that six of the eight proportional allocations that were accepted across all bargaining situations had been proposed by players 1 and 2. The details with respect to each location and each group at each location are gathered in S2 and S3 Files in the Supporting Information.

**Table 2:** Players 1–4 with a focus on proportionality.

Proposer (endowment in € or £)	Number of all proposals	Proportional division (% of total number)	Rejections of proportional division by				Acceptance of proportional division as final solution
			Player 1	Player 2	Player 3	Player 4	
**a) All rounds**							
Player 1 (5)	172	15 (8.7%)	-	10	3	0	4
Player 2 (10)	163	12 (7.4%)	7	-	3	3	2
Player 3 (15)	171	28 (16.4%)	26	21	-	6	0
Player 4 (20)	169	24 (14.2%)	16	16	9	-	2
*Total*	*675*	*79 (11*.*7%)*	*49*	*47*	*15*	*9*	*8*
**b) First four rounds only**							
Player 1 (5)	73	13 (17.8%)	-	8	1	0	4
Player 2 (10)	67	8 (11.9%)	5	-	3	2	2
Player 3 (15)	69	15 (21.7%)	14	9	-	1	0
Player 4 (20)	69	17 (24.6%)	11	10	5	-	2
*Total*	*278*	*53 (19*.*1%)*	*30*	*27*	*9*	*3*	*8*
**c) First round only**							
Player 1 (5)	24	9 (37.5%)	-	5	0	0	4
Player 2 (10)	23	4 (17.4%)	2	-	2	2	0
Player 3 (15)	25	7 (28.0%)	7	4	-	1	0
Player 4 (20)	15	5 (33.3%)	3	4	1	-	1
*Total*	*87*	*25 (28*.*7%)*	*12*	*13*	*3*	*3*	*5*

Given are absolute frequencies of all proposals, proposals in accordance with the proportionality principle, and rejections or acceptances of such proposals for (a) all rounds of all 90 groups, (b) the first four rounds, and (c) the very first round. Cases in which the proposer did not make any proposal within the two-minute time limit are excluded.

One may justly question the validity of the foregoing analysis on the grounds that the data points, while independent *across* groups of players, are not so *within* groups. A player may pick up an earlier proposal by one of his or her group members and just repeat this suggestion because they consider it a reasonable proposal. Alternatively, this player may, as a reaction to this earlier suggestion, propose a split of the loss, which is very different. When looking at all the data points at all locations, there are several instances where such an interpretation is fully justified.

On the other hand, one may argue that the phenomenon of possibly interdependent proposals simply mirrors a sequential procedure among the players who search for a common solution. And this is an interesting aspect which is characteristic of all bargaining situations but, in our view, holds *a fortiori* when the number of “contestants” increases beyond two.

With the above objection to our analysis so far in mind, we shall now examine how the proportionality principle fared in the first few rounds of bargaining and how this rule fared in the very first round. In the latter, the independence of observations is, of course, “naturally” preserved. Similar to our observation from Table 1, parts b) and c) of Table 2 show that the relative frequency with which proportionality is proposed increases considerably when only the number of proposals in the first four rounds or even in the very first round are considered. Notice that the first four rounds already determine all the instances in which proportionality was accepted as a final solution. What is perhaps surprising is the fact that the tableaus of individual rejections are, qualitatively speaking, very similar between parts a) and b).

The data suggests that many participants initially tested whether a proportional distribution would be acceptable to the other group members, and since this was apparently not the case, they then looked for other solutions. On the other hand, it is interesting to notice that the rejection rates of players 1 and 2 are relatively stable across the various rounds. As parts a)–c) of Table 2 demonstrate, the rejection rate of player 1 is 12/16 (75%) in the first round, 30/40 (75%) in the first four rounds and 49/64 (77%) across all rounds. For player 2, the corresponding proportions are 62% in the first round, 60% in the first four rounds, and 70% across all rounds, a slight increase.

We had asserted above that in relation to all bargaining situations players 1 and 2 propose a proportional share much less frequently than players 3 and 4. As part b) in Table 2 shows, this observation still holds with respect to the first four rounds but can no longer be upheld in relation to the very first round of proposals within each group. Somewhat surprisingly, in 9 out of 24 cases, when he or she was asked to make the first proposal, player 1 proposed the proportional solution.

When we look at the detailed information on proposals of proportional loss sharing presented in S2 File in the Supporting Information, another aspect of the decision process appears. Here, the total number of rounds per group and the round number when a proportional division was proposed are given. While 15 groups reached agreement already in the first round, others bargained much longer (with a maximum of 24 rounds). On average, it took participants in all 90 groups 7.62 rounds to agree on a solution or to reach the time limit of 20 minutes, while the corresponding median was 5.50. The average bargaining duration in the no-quiz treatment was 490/66 = 7.42 rounds (median 5.00), in the quiz treatment it was 196/24 = 8.17 rounds (median 8.50). Although a two-sample Wilcoxon rank-sum test indicates no differences between round numbers of both groups (p = 0.4361), this difference corroborates an observation in [*18*]. The authors found that with information about performance (and our players knew that the result in the quiz determined the distribution of initial endowments), tensions in entitlements increase the time before an agreement is reached.

Did a proposal of proportionality in the very first round shorten the bargaining procedure? There were 25 proposals with 160 rounds altogether so that the average number of rounds came up to 160/25 = 6.40, while the median value equals 4.00. This is lower than the average number across those bargaining situations, in which a different proposal has been made (496/62 = 8.00, median of 6.50), although the differences are not statistically significant (two-sample Wilcoxon rank-sum test, p = 0.2446). Additionally, we checked whether the length of bargaining within a group depends on who makes the proportionality proposal in the first rounds. We found that whenever player 1 makes such a proposal within the first four rounds, the bargaining length is the shortest among all players, both for the no-quiz treatment and the quiz treatment, whereas in case player 4 issues such a proposal, the bargaining period is the longest for both treatments. However, again differences are not statistically significant.

Let us next consider the issue of exemption. Part a) of Table 3 states all frequencies of proposals made by the different players, which exempt player 1 or players 1 and 2 simultaneously. Obviously, in almost 70% of all proposals suggested by player 1, the person with the lowest endowment is exempted from any additional burden. Likewise for those suggested by player 2. Furthermore, although the corresponding percentage value is significantly lower (two-sided Fisher’s exact test, p<0.001), about half of all proposals by players 3 and 4 also leave out player 1. Hence, an allocation, which omits player 1 from carrying any losses, is likely to be unanimously accepted. Things are a bit different for divisions, which also exclude player 2 from carrying any losses. While it is no surprise that about 47% of all proposals by player 2 belong to this group, on the average only 20.7% of the suggestions by the other three players leave out players 1 and 2 (p<0.001 according to Fisher’s exact test).

**Table 3:** Proposals exempting player 1 or players 1 and 2.

Proposer (endowment in € or £)	Total number of proposals	Proposals exempting player 1 (% of total number)	Proposals exempting players 1 and 2 (% of total number)
**a) All rounds**			
Player 1 (5)	172	118 (68.6%)	43 (25.0%)
Player 2 (10)	163	113 (69.3%)	76 (46.6%)
Player 3 (15)	171	76 (44.4%)	40 (23.4%)
Player 4 (20)	169	87 (51.4%)	23 (13.6%)
*Total*	675	394 (58.4%)	182 (27.0%)
**b) First four rounds only**			
Player 1 (5)	73	52 (71.2%)	18 (24.7%)
Player 2 (10)	67	47 (70.2%)	38 (56.7%)
Player 3 (15)	69	30 (43.5%)	14 (20.3%)
Player 4 (20)	69	33 (47.8%)	10 (14.5%)
Total	278	162 (58.3%)	80 (28.8%)
**c) First round only**			
Player 1 (5)	24	14 (58.3%)	4 (16.7%)
Player 2 (10)	23	14 (60.9%)	12 (52.2%)
Player 3 (15)	25	12 (48.0%)	6 (24.0%)
Player 4 (20)	15	7 (46.7%)	3 (20.0%)
*Total*	87	47 (54.0%)	25 (28.7%)

Given are absolute frequencies of all proposals and proposals that exempt player 1 or players 1 and 2 from carrying any losses for (a) all rounds of all 90 groups, (b) the first four rounds, and (c) the very first round. Cases in which the proposer did not make any proposal within the two-minute time limit are excluded.

Parts b) and c) of Table 3 show that in contrast to what we have witnessed in Table 2, the views about exempting player 1 and players 1 and 2, respectively, are relatively stable, almost independent of whether one considers all rounds, the first four rounds or only the very first round. Conformity is particularly large between the cases of all rounds and the first four rounds. However, the situation of the very first round is not far apart.

To validate the robustness of our descriptive results presented until now, we estimated various regression models summarized in Table 4. Due to potential biases from interdependent proposals within each group mentioned above, we focused our analysis on group-specific characteristics and considered first round proposals and final group outcomes in binary regression models and potential effects on the duration of the bargaining process in a zero-truncated Poisson regression model. In each case, we report average marginal effects.

**Table 4:** Average marginal effects of group characteristics on first round proposals, final decisions and durations.

	First round proposals (binary logit regression)			Final decision (binary logit regression)				Duration (Poisson regression)
	Proportional division	Exempting player 1	Exempting players 1 & 2	Reaching Agreement	Agreement: Proportional division	Agreement: Exempting player 1	Agreement: Exempting players 1 & 2	
Dependent variable	(1 = proportional 0 = otherwise)	(1 = exemption 0 = otherwise)	(1 = exemption 0 = otherwise)	(1 = agreement 0 = otherwise)	(1 = proportional 0 = otherwise)	(1 = exemption 0 = otherwise)	(1 = exemption 0 = otherwise)	(Number of rounds)
Quiz	0.26* (0.14)	-0.12 (0.15)	0.19 (0.16)	-0.14 (0.14)	0.01 (0.10)	-0.06 (0.15)	0.10 (0.14)	3.87* (2.30)
Experimenter	0.01 (0.12)	0.04 (0.13)	-0.18 (0.11)	0.28** (0.12)	0.04 (0.07)	0.10 (0.13)	-0.06 (0.13)	-3.57* (2.01)
Endowment:								
10	-0.23** (0.12)	0.03 (0.14)	0.36*** (0.12)	—	—	—	—	—
15	-0.11 (0.14)	-0.10 (0.14)	0.08 (0.11)	—	—	—	—	—
20	-0.16 (0.12)	-0.07 (0.16)	0.01 (0.14)	—	—	—	—	—
Rounds	—	—	—	—	-0.02 (0.15)	<-0.01 (0.01)	-0.01 (0.01)	—
Prop. in 1^st^ round	—	—	—	0.15* (0.08)	0.20** (0.09)	-0.31** (0.13)	-0.10 (0.11)	-1.89 (1.50)
Sample size	87	87	87	87	70	70	70	87
Mean dep. var.	0.287	0.540	0.287	0.805	0.114	0.686	0.271	7.540
Wald χ^2^	8.19	1.80	10.11*	11.63***	8.86*	6.94	1.13	6.50*
Pseudo R^2^	0.075	0.017	0.108	0.125	0.214	0.083	0.018	0.041

The table states average marginal effects (dy/dx) after estimating either binary logistics regression models (first round proposal, final decision) or zero-truncated Poisson regression models (duration). All models included a constant term. Delta method standard errors are reported in parentheses. Explanatory variables: Quiz (endowments based on random assignment (= 0) or quiz results (= 1)), Experimenter (decision in case of no agreement by random mechanism (= 0) or experimenter (= 1), endowment of proposer (5 is reference group), Rounds (number of rounds until final decision), Prop. in 1^st^ round (proportional division has been proposed in first round (= 1) or not (= 0)).

Levels of significance

* 10%

** 5%

*** 1%.

Cases in which the proposer did not make any proposal within the two-minute time limit are excluded.

In general, it can be observed that our main descriptive results are confirmed by these more advanced statistical tools. The fact that initial endowments are based on quiz results rather than on a random mechanism significantly increases the probability of observing proportionality proposals in the first round and also increases the duration of the bargaining process, which confirms earlier results [*18*] as already mentioned. However, there is no effect from the quiz on the final decision of the group. As expected, groups are more likely to reach an agreement and need fewer rounds of bargaining until the final decision if the experimenter is said to decide in case of no agreement rather than have a random mechanism take the decision. Nevertheless, this treatment displays no effect on the agreement reached. Regarding the endowment of first round proposer we can see that player 1 more often proposes the proportional division and that player 2 more frequently wants to exempt the two players with lowest endowment, which indicates a clear self-serving bias [39–41]. Furthermore, while the final decision in case of an agreement is not associated with the number of rounds played, the fact that a proportional split has been proposed in the very first round seems to lead to higher rates of agreement, more frequent proportional divisions and fewer exemptions of player 1 in the final decision. Additionally, the duration also declines in this case, but the effect is not statistically significant.

Finally, we are able to incorporate additional socio-demographic characteristics of proposers in some of our regression models to validate our previous results and to get further insights into potential effects from these variables. At the end of the experiment, each player was asked some questions on his or her socio-demographic background (see again S1 File and S2 Table in the Supporting Information). The mean age and the corresponding standard deviation reflect the fact that the sample comprises both bachelor and master students. Hence, age is a good proxy for the different stages of the students’ careers and their corresponding educational level. The statements of the perceived income of the family in which the respondent grew up are normally distributed over the seven response categories with a mean value close to the middle category. In contrast, many respondents expected to earn a rather high income. This seems to be a realistic view for student participants who are enrolled in business administration or economics programs. The mean political orientation of the participants is slightly more to the left, but it should also be noted that all response categories have been selected.

S3 Table in the Supporting Information reports estimated binary regression models for the very first round, while S4 Table considers all rounds keeping in mind the interdependence problem within groups described above. Let us focus only on the main results. Both male participants and students who reported a political orientation towards the right more often proposed the proportional division in the very first round, but less frequently solutions, which exempted player 1. This observation also holds for right-wingers in later rounds. Furthermore, the strong endowment effects on exempting players already observed in Table 3 are confirmed by the regression models for all rounds. Obviously, players 3 and 4 are much less likely to exempt player 1 or players 1 and 2 especially after round 1. Finally, from the results in S4 Table it can also be seen again that proportional divisions are significantly less often proposed in later rounds. Hence, in summary even after having controlled for several socio-demographic characteristics our main descriptive results hold.

## Alternative proposals for loss distribution

Having focused almost entirely on the issue of proportionality in the preceding sections, the reader may legitimately ask what the bargaining proposals of the majority of players actually looked like. Salient contestants are the principle of an equal split of the overall loss and the constrained equal awards (CEA) rule. For the given vector of initial endowments in our experiments, namely w = (5, 10, 15, 20), the first rule would lead to a loss vector of (2.5, 2.5, 2.5, 2.5), the second to a vector of losses (0, 0, 2.5, 7.5). Elsewhere [42], we proposed and axiomatically characterized a rule which takes an equal split of the loss plus a weighted difference between the initial endowment of each player and the average over all individual endowments as reference points. In what follows, we shall label this scheme as “alpha rule”, “alpha” being a weight that can be interpreted as a behavioral parameter. More formally, let L be the aggregate loss, n the number of players, w = (w_1_, w_2_, …, w_n_) the vector of initial endowments, and m(w) the average initial endowment. Then it is proposed that the individual contribution of player i to the overall loss, l_i_(w), is given by:

$$l_{i}\left(w\right)=L/n+\alpha \left(w_{i}-m\left(w\right)\right).$$

It is easy to see that an alpha value equal to zero would lead to an equal split of the loss, 𝛼 = 1/5 would generate the loss vector (1, 2, 3, 4), 𝛼 = ¼ would lead to (0.625, 1.875, 3.125, 4.375), and 𝛼 = 1/3 would generate the vector (0, 1 2/3, 3 1/3, 5). This shows that an equal split and a proportional share of the loss are special cases of the proposed rule. Since, as was demonstrated in this paper, a proportional share turned out to be highly unpopular, the CEA rule with a zero contribution by the two players with the two lowest endowments, and the total burden heaved on the better endowed may prove to be a serious (i.e. widely accepted) alternative. However, it may also be felt by the majority of players that a total exemption of two players would be “just too much” to accept as a general solution, so that the vector (0, 1 2/3, 3 1/3, 5), in some way a middle path between proportionality and CEA, may get wider support.

Since most of the participants in our experiments across all sites were undergraduates, only a few were graduate students, it is, we think, safe to assume that they were neither familiar with the alpha rule nor with the CEA scheme (they may have, of course, known the proportional rule and the equal share principle from every day experience). So it would be naïve to expect them to act precisely according to either rule. To allow for this, we borrowed an idea proposed in [*15*] of measuring the Euclidean distance between each individual proposal and the rules mentioned in this section. We could, of course, have used a different measure, the min norm or the max norm, for example, but we do not see any deeper reason why these norms would have been more justified in the present context. Then, for each individual proposal that was made, the “winner” among these rules was determined as the one with the smallest Euclidean distance. Table 5 shows the summary results across all treatments and all locations, again distinguishing between first round only, first four rounds and all rounds. For an alpha value of 1/3, the rule that was proposed proved to be a clear winner in relation to all rounds, also a winner with respect to the first four rounds. It was only beaten by the proportionality rule in relation to the first round, which again shows that proportionality was an attractive proposal at the very beginning of each game but significantly lost support during the bargaining process. For α = 1/3, the proposed scheme ranked first in terms of Euclidean distance in seven out of ten samples or sites where the experiments took place, while CEA was ranked first in only three instances (in Table 5, we have omitted details in relation to all ten sites). Once one extends this analysis by not only focusing on the winning position (first rank) but also on the second rank, the proposed rule with 𝛼 = ¼ which fared rather poorly as Euclidean winner, now comes powerfully to the fore. But note that also in terms of first rank frequencies, the alpha rule with a weight of ¼ received more support when the number of bargaining rounds went up (the same observation also holds true with respect to α = 1/3). The CEA rule, *par contre*, fares relatively poorly in terms of second rank. So it appears that some contribution by the two players with the smallest endowments but not as much as the proportional scheme would require, receives a significant amount of support in the eyes of the players. The proportional rule lies way behind in this rank-based perspective.

**Table 5:** A rank-based comparison among alternative rules.

	*α* = 0: Equal losses	*α* = 1/3	*α* = 1/4	*α* = 1/5: Proportional	CEA
**a) All rounds (n = 675)**					
1^st^ rank frequencies (%)	69 (10.2)	226 (33.5)	71 (10.5)	110 (16.3)	199 (29.5)
2^nd^ rank frequencies (%)	5 (0.7)	231 (34.2)	272 (40.3)	108 (16.0)	59 (8.7)
**b) First four rounds only (n = 278)**					
1^st^ rank frequencies (%)	27 (9.7)	83 (29.9)	24 (8.6)	63 (22.7)	81 (29.1)
2^nd^ rank frequencies (%)	3 (1.1)	91 (32.7)	118 (42.5)	41 (14.8)	25 (9.0)
**c) First round only (n = 87)**					
1^st^ rank frequencies (%)	11 (12.6)	24 (27.6)	2 (2.3)	28 (32.2)	22 (25.3)
2^nd^ rank frequencies (%)	2 (2.3)	23 (26.4)	45 (51.7)	12 (13.8)	5 (5.8)

## Final discussion

Multilateral bargaining games for situations in which a benefit or surplus has to be divided among a group of agents are manifold. Much less attention has been paid to multilateral bargaining over losses. In the introduction, we gave several examples, which demonstrate that the issue of loss sharing is highly relevant for economic and social policy. This lack of attention to the phenomenon of losses may be due to the conjecture that theoretical predictions of a multilateral divide-a-loss game would be exactly the inverse of those of a divide-a-benefit game. We think that this is not the case. As the authors in [*35*] argue, rather figuratively, comparing the divide-a-benefit game with the divide-a-loss game “is not analogous to comparing the allocation of a ‘half-full’ cup of water to that of a ‘half-empty’ cup; instead, it is analogous to comparing the allocation of a full cup of ‘clean’ water to that of a full cup of ‘filthy’ water” (p. 2). And they continue saying that “the former example, though framed differently, deals with the same objective, but the latter deals with fundamentally different objectives” (p. 2).

Our objective in this paper has been an experimental analysis of the loss-division problem with a focus on the role that the proportionality rule would play in such a context. Undoubtedly, the proportionality principle is an intuitive scheme, easy to understand and often applied in real-world liquidation situations [*29*]. So one might argue that proportionality has an *a priori* advantage over other rules when judged from outside. The authors in [*17*] argue that the inclination to opt for the proportional rule may be facilitated by some sort of median voter effect, as proportionality is somewhat situated in between extreme sharing rules such as an equal split of a loss on one side and a heavy burden on those who are amply endowed on the other, as constrained equal awards would suggest. The authors speak of the proportionality rule as a coordination device.

Our main result, however, is that proportionality was rarely proposed and even less frequently adopted as an acceptable solution. This finding robustly survived under different treatments and different locations (which was a big surprise to us). The participants’ rejection of the proportionality principle as a *solution* to our game of loss distribution has been overwhelming. These results are to some degree in conformity with findings in [*15*] where in a two-player situation with differing claims, the proportionality rule fared relatively badly. We say “to some degree”, because first of all free-form bargaining in [*15*] was between two players only. Furthermore, the agents were informed that if they failed to reach an agreement, they would earn nothing except for a show-up fee, an announcement which is quite different from the two treatments that we examined, namely a random choice over the players’ past proposals or a decision made by the experimenter, in case there were no unanimous agreement after 20 minutes. Secondly, the equal split solution received much support in [*15*], particularly in the case in which the claims diverged significantly, whereas in our investigations equal split clearly lost against our own scheme (with α = 1/3), constrained equal awards and proportionality, as documented in Table 5 above.

This brings us to another point worth discussing, namely the fact that results apparently vary quite a bit among different experimental set-ups. In [*17*], the proportionality principle overwhelmingly prevailed in a coordination game based on majority voting where three players had different claim points. These findings were strongly confirmed in [*19*] for a setting in which third-party arbitrators made decisions in a bankruptcy situation in which two agents had earned claims during a production phase. On the other hand, in a multilateral bargaining scenario among three and five agents respectively, with an equal endowment for all players, in which a proposer’s suggestion of how to divide a loss had to be approved by simple majority, an extreme allocation of the loss to a few members turned out to be the outcome [*35*]. Under the unanimity rule *par contre*, an almost equal split among the agents was observed, a finding that we had obtained in a few very early experiments for the case of equal endowments as well. The authors of [*22*] state that in two-person ultimatum games, outcomes close to equal split are more likely to be witnessed in the loss domain than they are in the gains context. In unstructured bargaining, namely scenarios in which both players simultaneously make their offers, it was found that equal split is the most common method of distribution in the loss domain [*43*], which is in stark contrast to our own experimental findings, though ours were within a sequential process of proposals. Finally, a non-negligible fraction of the arbitrators in [*19*] decided according to the constrained equal losses rule, with hardly any support for constrained equal awards, the dominant principle in [*15*].

Is it then possible to come up with some general verdict after these rather divergent findings? It is probably safe to say that proportionality is a widely accepted rule both in questionnaire experiments and third-party arbitrator decisions in the bargaining context, in particular when the issue is to distribute the liquidation value among claimants in the case of bankruptcy. The situations that we have considered differ substantially from these two scenarios. We did not use questionnaires to elicit judgments as in [*15–17*]. Empirical results derived from questionnaire studies are primarily meant to obtain information about norms and are therefore used as an input to normative discussions. These results can be quite different from the observation of actual behavior in certain competitive or even antagonistic situations where, as in our set-up, a smaller contribution of one agent automatically implies a larger contribution by other agents. In the majority procedure of [*17*], the participants who were in the role of depositors or creditors had to choose among three *given* rules that were introduced to them. The agents then had to play 20 rounds of the game they were participating in. Furthermore, the instruction in the majority game was that if only two of the agents agree on one of the given rules, those two obtain the share that this rule proposes while the third person who does not agree with this division not only loses his or her claim but also pays a fixed penalty. In such a situation, each agent may ponder over what is going on in the others’ mind. A decision in favor of the proportional rule as an easily intelligible scheme may then appear quite obvious. These features are totally absent from our set-up. As an aside, we would like to mention that in the majority games of [*17*] the frequency of coordination on the proportionality rule significantly increased in later rounds of the game, while in our experiments, as discussed above, the frequency of proposals of proportionality clearly decreased in a comparison between first round only and all rounds.

Coming back to several other of our findings, it is interesting to take a closer look at the no-quiz and quiz treatment and compare the proposals of player 4 with 20 units of money and player 1 with only 5 units, the polar cases, so to speak, as far as their initial endowments are concerned. The experiments in London in November 2017 and March 2018 when both treatments were run are revealing in this regard. We calculate the average proposals of players 1 and 4 for their own loss contribution at the group level. Two-sample Wilcoxon rank-sum tests are used to compare these values between the no quiz and quiz treatment. The first test includes 40 groups, in which player 4 makes a proposal, and reveals that this player offers significantly more often lower contributions for himself or herself after the quiz (p = 0.0277). So it seems plausible that this player was led by the idea that he or she deserved a smaller contribution because of his or her success in the quiz. Is this a self-serving characteristic or is it an expression or feeling of desert? Regarding the average proposals of player 1 in each of the 43 groups, in which he or she made a proposal, we find that higher loss contributions for himself or herself are more often proposed after the quiz (p = 0.0990). Apparently, player 1 finds that he or she should contribute more after a (weak) performance in the quiz, while player 4 should contribute somewhat less (p = 0.2714) which, however, is not significant. Player 4 also seems to think that player 1 should pay more after the quiz result (p = 0.0118). Granted, in relation to the status of a quiz in experiments, there is the issue of whether a quiz is a real effort task and if so, whether it is a legitimate measure of desert. Of course, we do not know. Given the above figures for the two polar cases, we would, however, argue that both players were willing to recognize both weak performance and success.

When we compare the treatment of a random choice among earlier proposals of the players, if there is no agreement at the end of the game, with the treatment that the decision maker decides about the final settlement, there is another interesting aspect that our findings reveal. Under the first treatment, the players were plausibly speculating that one of their own self-serving proposals might, with some “luck”, come out as the final loss distribution, much to their own advantage. Since this kind of thought may have occupied the mind of several players within a group, the sequence of proposals contained several quite unacceptable ones and rendered the bargaining duration considerably longer.

With respect to bargaining duration, we can state that in treatments in which the endowment assignment reflected success or desert, and this was, as the reader will remember, common knowledge among the group members, negotiations took considerably longer. This is in line with findings in [*18*]. Furthermore, a proposal of proportionality in the very first round shortened the bargaining duration but in no way led to general acceptance of this principle, except for a few cases in which such a proposal, issued by player 1, led to an early agreement. As we saw in Table 3, the structure of exemption in relation to player 1 and players 1 and 2, respectively, is surprisingly stable if one moves from proposals in all rounds to the case of four rounds and to the situation in the very first round. This fact seems to reflect a certain degree of consideration towards these two players.

Finally, we would like to re-iterate that bargaining over losses between two agents is quite different from situations with four players where distributional aspects come much more into prominence and where the dynamics of negotiation are much more pronounced. It is in this sense that our own results hopefully provide new insights so that they can be seen as complementary to earlier findings.

## Supporting information

S1 File Instructions to players. (PDF)

S2 File Proposals of proportional loss sharing at all sites. (PDF)

S3 File Group behavior in the first four rounds at all sites. (PDF)

S4 File Basic data set. (CSV)

S1 Table Fraction of proposals for proportionality within each group. (PDF)

S2 Table Socio-demographic characteristics. (PDF)

S3 Table Average marginal effects of group and individual characteristics on first round proposals. (PDF)

S4 Table Average marginal effects of group and individual characteristics on proposals in all rounds. (PDF)
