# Supplementary material for: Against the proportionality principle: Experimental findings on bargaining over losses
Source: PLoS One. 2019 Jul 22;14(7):e0218805. doi: 10.1371/journal.pone.0218805 (PMC6645459; doi:10.1371/journal.pone.0218805)
Supplement: S1 File — (PDF) [file pone.0218805.s001.pdf]

S1 File. Instructions to players

Verbal announcements before the experiment

- Thank you very much for your participation in this experiment.
- The experiment is part of a larger research project financed by the LSE on aspects of the allocation of losses.
- You will be asked to allocate a loss among you and three other randomly chosen participants here in the room.
- The experiment is anonymous. All information is anonymized and serves only scientific purposes.
- During the experiment it is not allowed to use electronic devices or to communicate with other participants. Please use only the programmes and functions provided by the experiment. Please do not talk to other participants.
- You will receive an instruction sheet.
- At the top of the sheet you will find a code number which is necessary to log-in to the program.
- The code number contains 8 small letters and 4 numbers without spaces.
- After the registration, you will have to go through these instructions, first.
- From time to time during the experiment you will need to wait while another participant is making a decision. Please remain silent while waiting.
- If you have any questions, please raise your hand. We will come to you. Please do not ask your question loudly.
- If you reach the final screen, please also remain silent and wait until all groups have finished the experiment.
- After the experiment you will receive your money in cash 10 minutes after the end of the experiment.

Experiment: Loss distribution

Welcome and thank you for participating in this experiment. Please read the instructions carefully. They are identical for all participants. The instructions are also made available to you in paper form. You are welcome to consult them at any time during the experiment.

- You are taking part in an experiment where, starting from an initial distribution of endowments in terms of real money, losses have to be shared.
- You will be assigned to a group of four. At no point in the experiment, your identity will be disclosed nor will the identities of your other group members be revealed.
- At the beginning of the experiment, an initial distribution of endowments will be determined and announced, specifying the amount of pounds that each member of your group receives.

[page break]

- Your group will have to share a **total loss of £10** that will have to be given back to the experimenter at the end of the experiment.

- One randomly chosen member of your group will make a first **proposal of how to distribute the loss among the members of your group.**
- This and every other proposal that may follow has to be made within two minutes.
- "Negative" losses are not acceptable nor are proposals for individual losses accepted that are higher than a person's initial endowment.
- This screenshot is an example for the input mask:

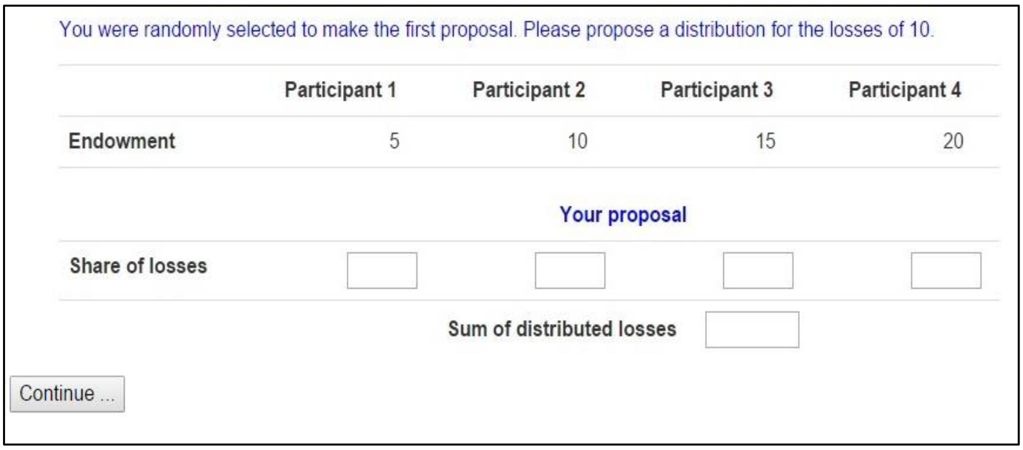

[page break]

- You are then asked to either **accept or reject this first proposal**. You have one minute to make your decision. Should you remain "silent", **this will be defined as acceptance.**
- If the first proposal is accepted by **all** members of your group, **the experiment is over** and you will be paid your final net amount in cash at the day of the experiment. You will receive an envelope with your personal code number on it.
- Should there be no agreement, a second person in your group (also determined by chance, but, of course, different from the first person) will have to make a proposal of how to split up the loss.
- As long as no agreement on one proposal is reached among the group members, a new participant is chosen at random to propose a distribution of the loss of £10.
- While a new proposer is always chosen randomly, no person will be asked to make a second proposal before all other members have made at least one proposal (proposers are drawn from a hat without replacing).
- This last point equally applies in following rounds: Each participant will only get the chance to make **his next** proposal, after all players have also made at least one additional proposal.

[page break]

- If, at any point, one proposal receives unanimous agreement, the experiment is over and each participant receives his/her corresponding net payout as described above.
- There is a 20 minutes time limit for the experiment. After each round the remaining time is stated. Once the time has run out, the current proposal **can still be made** and the votes on it **can still be taken.**
- Please notice that if after these 20 minutes, none of the past proposals received unanimous support, the experiment is over.
- In this case, the **experimenter** will take his own decision on how the loss of £10 is going to be shared. Payment will be made as indicated above.

- At the end of the experiment, you will be asked to answer a couple of questions which are also treated fully anonymously. Thank you for this as well and thank you again for participating in this experiment!

Finally we ask you for some personal information. Please keep in mind that the questionnaire is anonymous and your answers are only used for scientific purposes.

1. Gender: O female O male
2. Age: ________ years
3. How would you rate your family's income ten years ago?

   very poor — very rich

   O —— O —— O —— O —— O —— O —— O
4. How would you rate your own income prospects in ten years?

   very low — very high

   O —— O —— O —— O —— O —— O —— O
5. How would you rate your political view?

   left — right

   O —— O —— O —— O —— O —— O —— O

Experiment: Quiz version

Alternative wording of 3rd bullet point in **S2**

- At the beginning of the experiment, there will be a short **knowledge quiz**. The result in this quiz determines your **initial endowment** and that of all other members in your group. This distribution will be announced to each of you, specifying the amount of pounds that each of you receives.

Knowledge quiz to determine the initial endowment

Please answer the following questions. Do not consult your smartphone during the quiz.
This might, if detected, lead to an exclusion of all members belonging to your group.
Please enter integer numbers only! Thanks.

Questions in the experiment	(Right answer)
• In 2015, how many licensed drivers were in the United States (please state the number in millions)?	(218)
• How many cars have been sold worldwide in 2016 (in millions)?	(77.31)
• What is the weight of the Statue of Liberty in the US (in tons)?	(225)
• In which year was the Nobel prize in economics awarded for the first time?	(1969)
• What is the height of the Eiffel tower in Paris (in metres)?	(324)
• What is the distance between London and Rio/Brazil (in miles)?	(5761)
• How many aircraft movements (number of flights arriving and departing) were counted for Gatwick Airport in 2016?	(275,633)
• How many passengers were transported by Eurostar (the train connecting France and the UK) in 2016?	(10,011,337)
• How many people attended games of the English Premier League in the season 2016/17?	(13,612,316)
• How many percent of the total world's landmass is covered by Russia? (in percent)	(10.995)

* Please do answer all the questions. This is important for determining ranks within your group and then the assignment of initial endowments according to ranks.

Payment procedure, not stated in the experiment: In each question, participants could earn 1 to 4 points depending on the closeness of their answer to the correct answer. Participants were ranked according to the sum of points they received in all 10 questions. Finally, this ranking determined the initial endowments of 5, 10, 15, and 20 Pounds.
