# Supplementary material for: Against the proportionality principle: Experimental findings on bargaining over losses
Source: PLoS One. 2019 Jul 22;14(7):e0218805. doi: 10.1371/journal.pone.0218805 (PMC6645459; doi:10.1371/journal.pone.0218805)
Supplement: S2 File — (PDF) [file pone.0218805.s002.pdf]

S2 File. Proposals of proportional loss sharing at all sites

Remark: In very few cases, proposers did not make any proposal until they reached their two-minute time limit. These rounds are also counted in the following tables but have been excluded in the tables in the main text, since they did not represent any substantial proposal.

Halle 2015 [1]. Total: 43 rounds. Proportionality: 9 proposals. Proportionality accepted: 0

Session	Group	Total number of rounds	Proportional allocation proposed by player	Round of proposal	Rejected by player(s)
1	1	9	4	4	1
1	1	9	2	8	1
1	2	1	-	-	-
1	3	16	4	2	1
1	3	16	1	3	2
1	3	16	4	6	1, 2, 3
1	4	4	3	1	1
1	5	12	1	2	2
1	5	12	3	4	1,2
1	5	12	4	7	1, 2
1	6	1	-	-	-

Madrid 2015 [2]. Total: 44 rounds. Proportionality: 5 proposals. Proportionality accepted: 1

Session	Group	Total number of rounds	Proportional allocation proposed by player	Round of proposal	Rejected by player(s)
1	1	1	-	-	-
1	2	15	1	7	2, 3
1	3	4	3	1	1, 2
1	3	4	4	4	*accepted*
1	4	5	-	-	-
1	5	2	-	-	-
1	6	17	3	9	1, 2
1	6	17	3	14	1, 2

Galway 2015 [3]. Total: 56 rounds. Proportionality: 7 proposals. Proportionality accepted: 1

Session	Group	Total number of rounds	Proportional allocation proposed by player	Round of proposal	Rejected by player(s)
1	1	3	2	1	3
1	2	19	1	1	2
1	2	19	3	14	1, 2
1	3	7	4	2	1, 2, 3
1	4	5	4	4	1
1	5	7	-	-	-
1	6	1	1	1	*accepted*
1	7	14	4	8	3

Berlin 2016 [4]. Total: 43 rounds. Proportionality: 7 proposals. Proportionality accepted: 0

Session	Group	Total number of rounds	Proportional allocation proposed by player	Round of proposal	Rejected by player(s)
1	1	21	4	15	1, 2
1	2	20	3	1	1, 2, 4
1	2	20	3	8	1, 2, 4
1	2	20	3	12	1, 2, 4
1	2	20	3	13	1, 2, 4
1	2	20	3	19	1, 2, 4
1	2	20	3	22	1, 2, 4
1	3	1	-	-	-
1	4	1	-	-	-

Halle 2016 [5]. Total: 53 rounds. Proportionality: 2 proposals. Proportionality accepted: 0

Session	Group	Total number of rounds	Proportional allocation proposed by player	Round of proposal	Rejected by player(s)
1	1	7	-	-	-
1	2	11	-	-	-
1	3	2	3	1	1, 2
1	4	2	-	-	-
1	5	3	-	-	-
1	6	16	4	3	2
1	7	12	-	-	-

London July 2017 [6]. Total: 84 rounds. Proportionality: 5 proposals. Proportionality accepted: 0

Session	Group	Total number of rounds	Proportional allocation proposed by player	Round of proposal	Rejected by player(s)
1	1	8	4	4	2
1	2	24	4	6	1, 2
2	1	12	3	3	1, 2
2	2	20	-	-	-
2	3	2	-	-	-
3	1	6	-	-	-
3	2	9	4	3	1
3	3	3	2	1	4

London November 2017 [7 + 8]. Total: 204 rounds. Proportionality: 25 proposals. Proportionality accepted: 2. Sessions 1-5 without, sessions 6-8 with quiz

Session	Group	Total number of rounds	Proportional allocation proposed by player	Round of proposal	Rejected by player(s)
1	1	4	-	-	-
1	2	5	2	4	1
1	3	3	-	-	-
2	1	5	-	-	-
2	2	1	-	-	-
2	3	2	3	1	1
2	4	2	-	-	-
2	5	1	-	-	-
3	1	6	-	-	-
3	2	3	3	2	1, 2
3	3	9	-	-	-
3	4	20	4	4	1,2, 3
3	4	20	3	10	1, 2
3	4	20	4	14	1, 2, 3
3	5	3	-	-	-
4	1	3	2	1	1
4	2	23	2	4	1,3
4	3	2	-	-	-
4	4	10	4	4	1, 2
5	1	4	-	-	-
5	2	1	1	1	*accepted*
5	3	1	-	-	-
5	4	7	3	4	1, 2
6	1	2	1	1	2
6	2	14	3	3	1, 2
6	3	10	4	1	1, 2, 3
6	4	9	-	-	-
7	1	1	-	-	-
7	2	1	4	1	*accepted*
7	3	8	4	2	2, 3
7	4	2	-	-	-
7	5	9	3	3	1
8	1	7	1	1	2
8	1	7	3	6	2
8	2	3	-	-	-

London November 2017 [7 + 8] (continued). Total: 204 rounds. Proportionality: 25 proposals. Proportionality accepted: 2. Sessions 1-5 without, sessions 6-8 with quiz

Session	Group	Total number of rounds	Proportional allocation proposed by player	Round of proposal	Rejected by player(s)
8	3	11	4	1	1, 2
8	3	11	3	10	1, 2
8	4	2	1	1	2
8	5	10	2	1	1, 3, 4
8	5	10	4	2	1, 3
8	5	10	2	7	1

London March 2018 [9 + 10]. Total: 156 rounds. Proportionality: 19 proposals. Proportionality accepted: 4. Sessions 1-3 without, sessions 4-6 with quiz

Session	Group	Total number of rounds	Proportional allocation proposed by player	Round of proposal	Rejected by player(s)
1	1	10	3	3	1
1	1	10	2	10	*accepted*
1	2	1	1	1	*accepted*
2	1	1	-	-	-
2	2	14	2	4	1
2	2	14	3	10	1
3	1	3	1	2	3
3	2	5	1	1	2
3	3	15	4	6	2, 3
4	1	4	-	-	-
4	2	9	1	2	2
4	2	9	3	3	2
4	2	9	3	6	1, 2
4	3	10	2	1	4
4	3	10	2	8	4
4	4	1	1	1	*accepted*
5	1	20	-	-	-
5	2	6	4	1	1, 2
5	3	15	-	-	-
5	4	22	4	1	2
6	1	16	1	14	2
6	2	4	3	2	1
6	2	4	2	4	*accepted*
