# Supplementary material for: Against the proportionality principle: Experimental findings on bargaining over losses
Source: PLoS One. 2019 Jul 22;14(7):e0218805. doi: 10.1371/journal.pone.0218805 (PMC6645459; doi:10.1371/journal.pone.0218805)
Supplement: S3 File — (PDF) [file pone.0218805.s003.pdf]

S3 File. Group behaviour in the first four rounds at all sites

Halle 2015 [1]

group	proposal for 5, 10, 15, 20 in the first four rounds	proposer (5, 10, 15, 20) and type of proposal(prop)	reaction by 5, 10, 15, 20 0=acc; 1=rej	accepted by all players
1	no proposal	5	no reaction	
	no proposal	10	no reaction	
	3, 3, 2, 2	15	0, 1, 0, 0	no
	1, 2, 3, 4	20 prop	1, 0, 0, 0	no
2	4, 2, 2, 2	5	0, 0, 0, 0	yes
3	1, 2, 2, 5	15	0, 1, 0, 1	no
	1, 2, 3, 4	20 prop	1, 0, 0, 0	no
	1, 2, 3, 4	5 prop	0, 1, 0, 0	no
	0.5; 1.5; 2.5; 5.5	10	0, 0, 0, 1	no
4	1, 2, 3, 4	15 prop	1, 0, 0, 0	no
	0, 2, 3, 5	10	0, 0, 0, 1	no
	0.25; 2; 3.25; 4.5	5	0, 0, 0, 1	no
	0.5; 2; 3; 4.5	20	0 ,0 ,0, 0	yes
5	3, 3, 2, 2	20	1, 1, 0, 0	no
	1, 2, 3, 4	5 prop	0, 1, 0, 0	no
	0, 0, 3, 7	10	0, 0, 1, 1	no
	1, 2, 3, 4	15 prop	1, 1, 0, 0	no
6	0, 2, 3, 5	5	0, 0, 0, 0	yes

Madrid 2015 [2]

group	proposal for 5, 10, 15, 20 in the first four rounds	proposer (5, 10, 15, 20) and type of proposal(prop)	reaction by 5, 10, 15, 20 0=acc; 1=rej	accepted by all players
1	2, 4, 2, 2	10	0, 0, 0, 0	yes
2	0, 1, 3, 6	5	0, 1, 1, 1	no
	3, 3, 2, 2	15	1, 1, 0, 0	no
	2, 3, 3, 2	20	1, 1, 1, 0	no
	0, 2, 3, 5	10	0, 0, 1, 0	no
3	1, 2, 3, 4	15 prop	1, 1, 0, 0	no
	3; 2.5; 2.25; 2.25	10	1, 0, 0, 0	no
	0, 2, 3, 5	5	0, 1, 0, 0	no
	1, 2, 3, 4	20 prop	0, 0, 0, 0	yes
4	0, 10, 0, 0	20	1, 1, 0, 0	no
	0, 0, 0, 10	5	0, 1, 0, 0	no
	0, 0, 3, 7	10	0, 0, 0, 1	no
	0, 2.5; 2.5; 5	15	1, 1, 0, 0	no
5	0, 2, 3, 5	5	0, 1, 0, 0	no
	0, 0, 3, 7	10	0, 0, 0, 0	yes
6	0, 0, 0, 10	10	0, 0, 1, 1	no
	1; 1.5; 2.5; 5	5	0, 1, 0, 0	no
	1.25; 2; 2.75; 4	15	1, 1, 0, 0	no
	0, 1, 4, 5	20	0, 1, 1, 0	no

Galway 2015 [3]

group	proposal for 5, 10, 15, 20 in the first four rounds	proposer (5, 10, 15, 20) and type of proposal(prop)	reaction by 5, 10, 15, 20 0=acc; 1=rej	accepted by all players
1	1, 2, 3, 4	10 prop	0, 0, 1, 0	no
	1, 2, 2, 5	5	0, 1, 0, 0	no
	1, 1, 3, 5	15	0, 0, 0, 0	yes
2	1, 2, 3, 4	5 prop	0, 1, 0, 0	no
	0, 2, 3, 5	15	0, 1, 0, 1	no
	2, 4, 4, 0	20	1, 1, 1, 0	no
	0.5; 0.5; 3; 6	10	1, 0, 0, 1	no
3	3, 3, 1, 3	15	1, 1, 0, 1	no
	1, 2, 3, 4	20 prop	1, 1, 1, 0	no
	1, 1, 4, 4	5	0, 0, 1, 0	no
	0, 0, 3, 7	10	0, 0, 1, 1	no
4	3, 3, 1, 3	15	1, 1, 0, 0	no
	1, 1, 4, 4	10	1, 0, 1, 0	no
	no proposal	5	no reaction	
	1, 2, 3, 4	20 prop	1, 0, 0, 0	no
5	0, 0, 5, 5	20	1, 1, 1, 0	no
	0, 2, 3, 5	5	0, 1, 0, 0	no
	0, 0, 3, 7	10	0, 0, 1, 0	no
	no proposal	15	no reaction	
6	1, 2, 3, 4	5 prop	0, 0, 0, 0	yes
7	0, 0, 3, 7	10	0, 0, 1, 1	no
	0, 1, 4, 5	5	0, 0, 1, 0	no
	1, 1, 2, 6	15	1, 0, 0, 1	no
	1.5; 2; 3; 3.5	20	1, 0, 1, 0	no

Berlin 2016 [4]

group	proposal for 5, 10, 15, 20 in the first four rounds	proposer (5, 10, 15, 20) and type of proposal(prop)	reaction by 5, 10, 15, 20 0=acc; 1=rej	accepted by all players
1	0, 0, 0, 10	10	0, 0, 0, 1	no
	0, 1, 3, 6	5	0, 1, 0, 1	no
	0, 1, 2, 7	15	0, 1, 0, 1	no
	0, 3, 3, 4	20	0, 1, 0, 0	no
2	1, 2, 3, 4	15 prop	1, 1, 0, 1	no
	1, 3, 4, 2	20	1, 1, 1, 0	no
	0, 0, 4, 6	5	0, 0, 1, 1	no
	0, 0, 3, 7	10	0, 0, 1, 1	no
3	0, 2, 3, 5	15	0, 0, 0, 0	yes
4	0; 0; 2.5; 7.5	5	0, 0, 0, 0	yes

Halle 2016 [5]

group	proposal for 5, 10, 15, 20 in the first four rounds	proposer (5, 10, 15, 20) and type of proposal(prop)	reaction by 5, 10, 15, 20 0=acc; 1=rej	accepted by all players
1	0; 0; 2.5; 7.5	10	0, 0, 0, 1	no
	0; 0.5; 3; 6.5	5	0, 0, 1, 0	no
	0.75; 1.75; 2.5; 5	15	0, 1, 0, 0	no
	1, 1, 2, 6	20	1, 1, 0, 0	no
2	0, 0, 3, 7	5	0, 0, 1, 1	no
	0, 1, 4, 5	15	0, 1, 0, 0	no
	0; 1.5; 3; 5.5	20	0, 1, 1, 0	no
	0; 0; 2.5; 7.5	10	0, 0, 0, 1	no
3	1, 2, 3, 4	15 prop	1, 1, 0, 0	no
	0, 1, 3, 6	20	0, 0, 0, 0	yes
4	5, 1, 1, 3	15	1, 1, 0, 0	no
	0, 0, 3, 7	10	0, 0, 0, 0	yes
5	0, 2, 3, 5	20	0, 1, 0, 0	no
	0.5; 2; 3; 4.5	5	0, 1, 0, 0	no
	0, 0, 3, 7	10	0, 0, 0, 0	yes
6	0, 2, 3, 5	5	0, 1, 0, 1	no
	0, 0, 0, 10	10	0, 0, 0, 1	no
	1, 2, 3, 4	20 prop	0, 1, 0, 0	no
	0; 0; 2.5; 7.5	15	0, 0, 0, 1	no
7	0, 0, 3, 7	10	1, 0, 0, 0	no
	0, 10, 0, 0	5	0, 1, 0, 0	no
	0, 1, 2, 7	15	1, 0, 0, 0	no
	0, 1, 3, 6	20	1, 0, 1, 0	no

London July 2017, no quiz [6]

group	proposal for 5, 10, 15, 20 in the first four rounds	proposer (5, 10, 15, 20) and type of proposal(prop)	reaction by 5, 10, 15, 20 0=acc; 1=rej	accepted by all players
1	0, 0, 3, 7	10	0, 0, 1, 1	no
	0, 1, 4, 5	5	0, 1, 1, 0	no
	2, 2, 3, 3	15	0, 1, 0, 0	no
	1, 2, 3, 4	20 prop	0, 1, 0, 0	no
2	0, 0, 3, 7	5	0, 0, 1, 1	no
	5, 5, 0, 0	15	1, 1, 0, 0	no
	2, 2, 4, 2	20	1, 1, 1, 0	no
	5, 0, 2, 3	10	1, 0, 0, 0	no
3	0, 0, 3, 7	10	0, 0, 1, 1	no
	0; 0; 2.5; 7.5	5	0, 0, 1, 1	no
	1, 2, 3, 4	15 prop	1, 1, 0, 0	no
	0.5; 1.5; 3; 5	20	1, 1, 0, 0	no
4	0, 0, 5, 5	5	0, 0, 1, 1	no
	1, 2, 2, 5	15	1, 1, 0, 1	no
	0.5; 1.5; 3.5; 4.5	20	1, 1, 1, 0	no
	0, 0, 5, 5	10	0, 0, 1, 1	no
5	2, 4, 2, 2	10	1, 0, 0, 1	no
	0, 0, 2, 8	15	0, 0, 0, 0	yes
6	no proposal	10	no reaction	
	0; 2.5; 3.5; 4	5	0, 1, 0, 0	no
	0, 0, 3, 7	15	0, 1, 0, 1	no
	0, 2, 3, 5	20	0, 1, 0, 0	no
7	0, 2, 4, 4	5	0, 0, 1, 1	no
	0; 0; 2.5; 7.5	15	0, 0, 0, 1	no
	1, 2, 3, 4	20 prop	1, 0, 0, 0	no
	1, 2, 2, 5	10	1, 0, 0, 1	no
8	1, 2, 3, 4	10 prop	0, 0, 0, 1	no
	0, 1, 4, 5	15	0, 1, 0, 0	no
	0, 1, 2, 7	20	0, 0, 0, 0	yes

London Nov. 2017, no quiz [7]

group	proposal for 5, 10, 15, 20 in the first four rounds	proposer (5, 10, 15, 20) and type of proposal(prop)	reaction by 5, 10, 15, 20 0=acc; 1=rej	accepted by all players
1	2, 2, 2, 4	10	1, 0, 0, 0	no
	0, 2, 3, 5	5	0, 1, 1, 0	no
	2, 2, 2, 4	15	1, 0, 0, 0	no
	0, 2, 2, 6	20	0, 0, 0, 0	yes
2	0, 1, 4, 5	5	0, 0, 1, 0	no
	0, 0, 5, 5	15	0, 0, 0, 1	no
	1, 1, 4, 4	20	1, 0, 0, 0	no
	1, 2, 3, 4	10 prop	0, 0, 0, 0	yes
3	0, 2, 0, 8	15	1, 1, 0, 1	no
	0; 1; 2.5; 6.5	20	1, 0, 1, 0	no
	0, 1, 2, 7	5	0, 0, 0, 0	yes
4	0, 2, 3, 5	10	1, 0, 0, 0	no
	0, 0, 3, 7	5	0, 0, 0, 1	no
	0, 0, 5, 5	15	0, 1, 0, 0	no
	0, 2, 2, 6	20	0, 1, 0, 0	no
5	0, 2, 3, 5	5	0, 0, 0, 0	yes
6	1, 2, 3, 4	15 prop	1, 0, 0, 0	no
	0, 1, 3, 6	20	0, 0, 0, 0	yes
7	0; 1.5; 2.5; 6	15	0, 1, 0, 0	no
	0, 1, 2, 7	10	0, 0, 0, 0	yes
8	0, 0, 5, 5	20	0, 0, 0, 0	yes
9	0; 0; 2.5; 7.5	10	1, 0, 0, 1	no
	0; 2.5; 2.5; 5	5	0, 1, 0, 0	no
	0; 0; 2.5; 7.5	15	1, 0, 0, 1	no
	0, 0, 3, 7	20	1, 0, 1, 0	no
10	0, 1, 3, 6	5	0, 1, 0, 0	no
	1, 2, 3, 4	15 prop	1, 1, 0, 0	no
	0, 0, 3, 7	20	0, 0, 0, 0	yes
11	0, 0, 3, 7	15	1, 0, 0, 1	no
	0; 1; 3.5; 5.5	20	1, 0, 0, 0	no
	0; 0; 2.5; 7.5	5	0, 0, 1, 1	no
	0, 0, 3, 7	10	1, 0, 1, 1	no
12	2.5; 2.5; 2.5; 2.5	15	1, 1, 0, 0	no
	0, 0, 2, 8	10	0, 0, 0, 1	no
	0, 2, 3, 5	5	0, 1, 1, 0	no
	1, 2, 3, 4	20 prop	1, 1, 1, 0	no
13	0, 0, 3, 7	20	0, 0, 1, 0	no
	0; 0; 2.5; 7.5	10	0, 0, 0, 0	yes
14	1, 2, 3, 4	10 prop	1, 0, 0, 0	no
	0, 0, 2, 8	5	0, 0, 0, 1	no
	0, 2, 3, 5	15	0, 0, 0, 0	yes

London Nov. 2017, no quiz [7] (continued)

group	proposal for 5, 10, 15, 20 in the first four rounds	proposer (5, 10, 15, 20) and type of proposal(prop)	reaction by 5, 10, 15, 20 0=acc; 1=rej	accepted by all players
15	0, 1, 3, 6	5	0, 0, 1, 0	no
	0, 2, 0, 8	15	1, 1, 0, 1	no
	0, 0, 3, 7	20	0, 0, 1, 0	no
	1, 2, 3, 4	10 prop	1, 0, 1, 0	no
16	0; 2.5; 2.5; 5	15	0, 1, 0, 0	no
	0, 2, 3, 5	20	0, 0, 0, 0	yes
17	0, 2, 3, 5	15	0, 1, 0, 1	no
	0, 1, 2, 7	10	0, 0, 0, 1	no
	0, 1, 3, 6	5	0, 0, 0, 1	no
	1, 2, 3, 4	20 prop	1, 1, 0, 0	no
18	0.25; 0.75; 3; 6	10	1, 0, 1, 0	no
	0, 1, 3, 6	5	0, 1, 1, 0	no
	0.5; 1.3; 2.7; 5.5	15	1, 1, 0, 0	no
	0; 0.75; 2.7; 6.55	20	0, 0, 0, 0	yes
19	1, 2, 3, 4	5 prop	0, 0, 0, 0	yes
20	0, 1, 3, 6	15	0, 0, 0, 0	yes
21	1, 2, 3, 4	15 prop	1, 1, 0, 0	no
	0, 0, 2, 8	10	0, 0, 0, 1	no
	0, 0, 4, 6	5	0, 0, 1, 0	no
	0, 0, 3, 7	20	0, 0, 1, 0	no

London Nov. 2017, quiz [8]

group	proposal for 5, 10, 15, 20 in the first four rounds	proposer (5, 10, 15, 20) and type of proposal(prop)	reaction by 5, 10, 15, 20 0=acc; 1=rej	accepted by all players
1	1, 2, 3, 4	5 prop	0, 1, 0, 0	no
	0; 0; 2.5; 7.5	10	0, 0, 0, 0	yes
2	0, 0, 5, 5	10	0, 0, 1, 1	no
	0; 1.5; 4; 4.5	5	0, 1, 1, 1	no
	1, 2, 3, 4	15 prop	1, 1, 0, 0	no
	0.5; 2; 3; 4.5	20	0, 1, 0, 0	no
3	1, 2, 3, 4	20 prop	1, 1, 1, 0	no
	0; 0; 2.5; 7.5	15	0, 0, 0, 1	no
	0; 0; 2.6; 7.4	10	0, 0, 0, 1	no
	0, 0, 3, 7	5	0, 0, 0, 1	no
4	0, 0, 3, 7	15	0, 0, 0, 1	no
	0, 2, 3, 5	20	1, 1, 0, 0	no
	0, 1, 3, 6	10	0, 0, 0, 1	no
	0, 0, 0, 10	5	0, 0, 0, 1	no
5	0, 2, 3, 5	10	0, 0, 0, 0	yes
6	1, 2, 3, 4	20 prop	0, 0, 0, 0	yes
7	0; 0; 2.5; 7.5	15	0, 0, 0, 1	no
	1, 2, 3, 4	20 prop	0, 1, 1, 0	no
	0, 0, 3, 7	10	0, 0, 0, 1	no
	2.5; 2.5; 2.5; 2.5	5	0, 1, 0, 0	no
8	0, 0, 0, 10	15	0, 0, 0, 1	no
	0, 0, 3, 7	5	0, 0, 0, 0	yes
9	0, 0, 3, 7	10	1, 0, 1, 0	no
	0; 0; 2.5; 7.5	5	0, 0, 1, 0	no
	1, 2, 3, 4	15 prop	1, 0, 0, 0	no
	0, 1, 2, 7	20	1, 0, 0, 0	no
10	1, 2, 3, 4	5 prop	0, 1, 0, 0	no
	2.5; 2.5; 2.5; 2.5	20	1, 0, 0, 0	no
	2, 3, 3, 2	10	1, 0, 1, 0	no
	0, 2, 3, 5	15	0, 1, 0, 0	no
11	0, 2, 3, 5	20	1, 0, 0, 0	no
	0, 0, 0, 10	10	1, 0, 0, 1	no
	0, 1, 3, 6	5	0, 0, 0, 0	yes
12	1, 2, 3, 4	20 prop	1, 1, 0, 0	no
	0, 1, 3, 6	10	0, 0, 0, 1	no
	0; 0; 3.5; 6.5	15	0, 0, 0, 1	no
	0; 0.5; 3.2; 6.3	5	0, 0, 0, 1	no
13	1, 2, 3, 4	5 prop	0, 1, 0, 0	no
	0; 0; 2.5; 7.5	20	0, 0, 0, 0	yes
14	1, 2, 3, 4	10 prop	1, 0, 1, 1	no
	1, 2, 3, 4	20	1, 0, 1, 0	no
	1.5; 2; 2.75; 3.75	15	1, 0, 0, 0	no
	0.5; 1.6; 3.4; 4.5	5	0, 0, 1, 1	no

London March 2018, no quiz [9]

group	Proposal for 5, 10, 15, 20 in the first four rounds	proposer (5, 10, 15, 20) and type of proposal(prop)	reaction by 5, 10, 15, 20 0=acc; 1=rej	accepted by all players
1	2, 1, 3, 4	10	1, 0, 1, 0	no
	0; 2.5; 3.5; 4	5	0, 1, 1, 0	no
	1, 2, 3, 4	15 prop	1, 0, 0, 0	no
	0, 0, 5, 5	20	0, 0, 1, 0	no
2	1, 2, 3, 4	5 prop	0, 0, 0, 0	yes
3	0, 0, 2, 8	10	0, 0, 0, 0	yes
4	0; 2.5; 2.5; 5	5	0, 1, 0, 0	no
	1; 2.5; 2.5; 4	15	1, 1, 0, 0	no
	0, 2, 3, 5	20	0, 0, 1, 0	no
	1, 2, 3, 4	10 prop	1, 0, 0, 0	no
5	2, 2, 3, 3	10	1, 0, 1, 0	no
	1, 2, 3, 4	5 prop	0, 0, 1, 0	no
	1, 1, 2, 6	15	0, 0, 0, 0	yes
6	1, 2, 3, 4	5 prop	0, 1, 0, 0	no
	1, 1, 2, 6	15	0, 0, 0, 1	no
	0, 2, 3, 5	20	0, 1, 0, 0	no
	0, 0, 5, 5	10	0, 0, 1, 0	no
7	no proposal	5	no reaction	
	0.75; 1.5; 2.75; 5	20	0, 1, 0, 0	no
	no proposal	15	no reaction	
	0; 0; 2.5; 7.5	10	0, 0, 0, 1	no

London March 2018, quiz [10]

group	Proposal for 5, 10, 15, 20 in the first four rounds	proposer (5, 10, 15, 20) and type of proposal(prop)	reaction by 5, 10, 15, 20 0=acc; 1=rej	accepted by all players
1	0; 0; 3.5; 6.5	15	0, 1, 0, 1	no
	0, 1, 4, 5	20	0, 1, 0, 0	no
	0; 0; 3.3; 6.7	10	0, 0, 1, 1	no
	0; 1; 3.5; 5.5	5	0, 0, 0, 0	yes
2	1, 3, 3, 3	20	0, 1, 0, 0	no
	1, 2, 3, 4	5 prop	0, 1, 0, 0	no
	1, 2, 3, 4	15 prop	0, 1, 0, 0	no
	0, 0, 3, 7	10	0, 0, 0, 1	no
3	1, 2, 3, 4	10 prop	0, 0, 0, 1	no
	3, 2, 1, 4	15	0, 0, 0, 1	no
	2.5; 2.5; 2.5; 2.5	20	0, 0, 1, 0	no
	0, 2, 3, 5	5	0, 0, 1, 1	no
4	1, 2, 3, 4	5 prop	0, 0, 0, 0	yes
5	0, 0, 0, 10	15	0, 0, 0, 1	no
	0, 0, 0, 10	10	0, 0, 0, 1	no
	0, 0, 3, 7	5	0, 0, 1, 1	no
	0.5; 0.5; 2, 7	20	1, 1, 1, 0	no
6	1, 2, 3, 4	20 prop	1, 1, 0, 0	no
	0, 2, 3, 5	15	0, 1, 0, 1	no
	0, 0, 4, 6	10	0, 0, 1, 1	no
	0, 2, 4, 4	5	0, 1, 1, 0	no
7	0, 0, 3, 7	10	0, 0, 0, 1	no
	0; 0; 2.5; 7.5	5	0, 0, 0, 1	no
	0, 1, 3, 6	15	0, 1, 0, 1	no
	1; 2; 2.5; 4.5	20	1, 1, 0, 0	no
8	1, 2, 3, 4	20 prop	0, 1, 0, 0	no
	1, 1, 3, 5	15	0, 1, 0, 0	no
	0, 2, 4, 4	5	0, 1, 1, 0	no
	0; 0; 2.5; 7.5	10	0, 0, 0, 1	no
9	0, 2, 3, 5	20	0, 1, 1, 0	no
	0, 1, 3, 6	10	0, 0, 1, 0	no
	0; 1.5; 3.5; 5	5	0, 0, 1, 0	no
	2, 2, 1, 5	15	0, 1, 0, 0	no
10	2.5; 2.5; 2.5; 2.5	20	1, 1, 0, 0	no
	1, 2, 3, 4	15 prop	1, 0, 0, 0	no
	0, 0, 4, 6	5	0, 0, 1, 0	no
	1, 2, 3, 4	10 prop	0, 0, 0, 0	yes
