# Supplementary material for: Against the proportionality principle: Experimental findings on bargaining over losses
Source: PLoS One. 2019 Jul 22;14(7):e0218805. doi: 10.1371/journal.pone.0218805 (PMC6645459; doi:10.1371/journal.pone.0218805)
Supplement: S1 Table — (PDF) [file pone.0218805.s005.pdf]

S1 Table. Fraction of proposals for proportionality within each group

Site	Group	Fraction of proposals for proportionality among all proposals [a]
Halle [1]	1	2/7
	2	0/1
	3	3/16
	4	1/4
	5	3/11
	6	0/1
Madrid [2]	1	0/1
	2	1/15
	3	2/4
	4	0/5
	5	0/2
	6	2/17
Galway [3]	1	1/3
	2	2/19
	3	1/7
	4	1/4
	5	0/6
	6	1/1
	7	1/14
Berlin [4]	1	1/21
	2	6/23
	3	0/1
	4	0/1
Halle [5]	1	0/7
	2	0/11
	3	1/2
	4	0/2
	5	0/3
	6	1/16
	7	0/12
London [6]	1	1/8
	2	1/23
	3	1/12
	4	0/20
	5	0/2
	6	0/6
	7	1/9
	8	1/3
London [7]	1	0/4
	2	1/5
	3	0/3
	4	0/5
	5	0/1
	6	1/2
	7	0/2

Site	Group	Fraction of proposals for proportionality among all proposals [a]
London [7] cont.	8	0/1
	9	0/6
	10	1/3
	11	0/9
	12	3/20
	13	0/2
	14	1/3
	15	1/23
	16	0/2
	17	1/10
	18	0/4
	19	1/1
	20	0/1
	21	1/7
London [8]	1	1/2
	2	1/14
	3	1/10
	4	0/9
	5	0/1
	6	1/1
	7	1/8
	8	0/2
	9	1/9
	10	2/7
	11	0/3
	12	2/11
	13	1/2
	14	3/10
London [9]	1	2/10
	2	1/1
	3	0/1
	4	2/13
	5	1/3
	6	1/5
	7	1/13
London [10]	1	0/4
	2	3/9
	3	2/10
	4	1/1
	5	0/20
	6	1/6
	7	0/15
	8	1/22
	9	1/16
	10	2/4

[a] Cases in which the proposer did not make any proposal within the time limit are excluded.
