# Supplementary material for: Against the proportionality principle: Experimental findings on bargaining over losses
Source: PLoS One. 2019 Jul 22;14(7):e0218805. doi: 10.1371/journal.pone.0218805 (PMC6645459; doi:10.1371/journal.pone.0218805)
Supplement: S2 Table — (PDF) [file pone.0218805.s006.pdf]

S2 Table. Socio-demographic characteristics

	Mean	Std. Dev.	Min	Max
Age (in years)	22.953	4.223	17	53
Sex (0=female, 1=male)	0.508		0	1
Family income (1=very poor, 7 = very rich)	4.172	1.172	1	7
Low (answers 1-3)	0.269		0	1
Middle (answer 4)	0.319		0	1
High (answers 5-7)	0.411		0	1
Future income (1=very low, 7 = very high)	5.194	1.095	1	7
Low (answers 1-3)	0.053		0	1
Middle (answer 4)	0.175		0	1
High (answers 5-7)	0.772		0	1
Political orientation (1=left, 7 = right)	3.711	1.269	1	7
Left (answers 1-3)	0.411		0	1
Middle (answer 4)	0.356		0	1
Right (answers 5-7)	0.233		0	1
Places [Samples] (Dummy coding)				
Halle [1]	0.067		0	1
Madrid [2]	0.067		0	1
Galway [3]	0.078		0	1
Berlin [4]	0.044		0	1
Halle [5]	0.078		0	1
London [6-10]	0.667		0	1

Number of observations: n = 360.
