# Supplementary material for: Against the proportionality principle: Experimental findings on bargaining over losses
Source: PLoS One. 2019 Jul 22;14(7):e0218805. doi: 10.1371/journal.pone.0218805 (PMC6645459; doi:10.1371/journal.pone.0218805)
Supplement: S3 Table — (PDF) [file pone.0218805.s007.pdf]

S3 Table. Average marginal effects of group and individual characteristics on first round proposals

	First round proposals (binary logit regression)		
	**Proportional division**	**Exempting player 1**	**Exempting players 1 & 2**
**Dependent variable**	(1=proportional, 0=otherwise)	(1=exemption, 0=otherwise)	(1=exemption, 0=otherwise)
Quiz	0.220* (0.115)	-0.149 (0.140)	0.117 (0.174)
Experimenter	-0.015 (0.101)	0.123 (0.121)	-0.082 (0.115)
Endowment (ref. 5):			
10	-0.179* (0.098)	0.012 (0.126)	0.368*** (0.112)
15	0.053 (0.121)	-0.216 (0.135)	0.053 (0.113)
20	-0.029 (0.120)	-0.160 (0.172)	-0.006 (0.158)
Socio-demographics:			
Age (in years)	-0.006 (0.11)	0.015 (0.013)	0.019* (0.011)
Sex (0=female, 1=male)	0.309*** (0.084)	-0.274*** (0.106)	-0.010 (0.096)
Family income (ref. low):			
Middle (answer 4)	-0.009 (0.107)	-0.023 (0.136)	-0.078 (0.116)
High (answers 5-7)	-0.095 (0.097)	-0.042 (0.134)	-0.126 (0.104)
Future income (ref. low, middle):			
High (answer 5-7)	-0.240* (0.128)	0.200 (0.138)	0.119 (0.111)
Political orientation (ref. left):			
Middle (answer 4)	0.073 (0.104)	-0.028 (0.122)	-0.043 (0.092)
Right (answers 5-7)	0.250** (0.112)	-0.255** (0.123)	-0.175 (0.107)
Sample size	87	87	87
Mean dep. var.	0.287	0.540	0.287
Wald χ^2	20.48*	15.02	16.30
Pseudo R^2	0.259	0.118	0.165

The table states average marginal effects (dy/dx) after estimating binary logistics regression models. Models included a constant term. Delta method standard errors are reported in parentheses. Explanatory variables: Quiz (endowments based on random assignment (=0) or quiz results (=1)), Experimenter (decision in case of no agreement by random mechanism (=0) or experimenter (=1), Endowment of proposer (5 is reference group). Base case of socio-demographics: female, low family income (answers 1-3), low or middle future income (answers 1-4), left political orientation (answers 1-3). Levels of significance: * 10%, ** 5%, *** 1%. Cases in which the proposer did not make any proposal within the two-minute time limit are excluded.
