# Supplementary material for: Against the proportionality principle: Experimental findings on bargaining over losses
Source: PLoS One. 2019 Jul 22;14(7):e0218805. doi: 10.1371/journal.pone.0218805 (PMC6645459; doi:10.1371/journal.pone.0218805)
Supplement: S4 Table — (PDF) [file pone.0218805.s008.pdf]

S4 Table. Average marginal effects of group and individual characteristics on proposals in all rounds

	**All rounds (binary logit regression)**		
	**Proportional division**	**Exempting player 1**	**Exempting players 1 & 2**
**Dependent variable**	(1=proportional, 0=otherwise)	(1=exemption, 0=otherwise)	(1=exemption, 0=otherwise)
Quiz	-0.027 (0.032)	-0.091 (0.081)	0.035 (0.078)
Experimenter	-0.003 (0.031)	0.083 (0.080)	-0.053 (0.066)
Number of proposal (ref. 1):			
2	-0.123*** (0.023)	-0.019 (0.042)	-0.046 (0.038)
3	-0.126*** (0.028)	0.006 (0.059)	0.001 (0.044)
4	-0.081* (0.041)	0.023 (0.076)	-0.051 (0.055)
5	-0.163*** (0.026)	0.143 (0.087)	-0.087 (0.090)
6	-0.137*** (0.031)	0.276*** (0.055)	0.016 (0.073)
Prop. in 1st round	0.138*** (0.028)	-0.062 (0.063)	-0.060 (0.058)
Endowment (ref. 5):			
10	-0.017 (0.030)	0.005 (0.058)	0.210*** (0.069)
15	0.090*** (0.034)	-0.256*** (0.059)	-0.046 (0.049)
20	0.055 (0.035)	-0.176*** (0.056)	-0.122** (0.062)
Socio-demographics:			
Age (in years)	0.004 (0.003)	-0.003 (0.007)	0.001 (0.005)
Sex (0=female, 1=male)	0.051* (0.028)	0.022 (0.055)	0.021 (0.055)
Family income (ref. low):			
Middle	0.019 (0.036)	-0.055 (0.067)	-0.058 (0.050)
High	-0.042 (0.030)	-0.042 (0.064)	-0.036 (0.046)
Future income (ref. low, middle):			
High	-0.070* (0.037)	0.146** (0.062)	0.029 (0.055)
Political orientation (ref. left):			
Middle	-0.004 (0.032)	-0.037 (0.051)	-0.082 (0.054)
Right	0.058* (0.032)	-0.202*** (0.068)	-0.133** (0.052)
Sample size	650	650	650
Mean dep. var.	0.117	0.588	0.269
Wald χ^2	170.80***	89.93***	54.08***
Pseudo R^2	0.179	0.091	0.087

The table states average marginal effects (dy/dx) after estimating binary logistics regression models with clustered standard errors at the group level and including a constant term. Robust Delta method standard errors are reported in parentheses. Explanatory variables: Quiz (endowments based on random assignment (=0) or quiz results (=1)), Experimenter (decision in case of no agreement by random mechanism (=0) or experimenter (=1)), Number of proposals (by this player), Prop. in 1st round (proportional division has been proposed in first round (=1) or not (=0)). Endowment of proposer (5 is reference group). Base case of socio-demographics: female, low family income (answers 1-3), low or middle future income (answers 1-4), left political orientation (answers 1-3). Levels of significance: * 10%, ** 5%, *** 1%. Cases in which the proposer did not make any proposal within the two-minute time limit are excluded.
